# Synergistic Antitumor Effects of Anlotinib Combined with Oral 5-Fluorouracil/S-1 via Inhibiting Src/AKT Signaling Pathway in Small-Cell Lung Cancer

**DOI:** 10.1155/2022/4484211

**Published:** 2022-06-16

**Authors:** Xinhang Xia, Wenhu Pi, Yanli Lan, Xiaomai Wu, Dongqing Lv, Yinnan Meng, Haihua Yang, Wei Wang

**Affiliations:** ^1^Key Laboratory of Radiation Oncology of Taizhou, Radiation Oncology Institute of Enze Medical Health Academy, Department of Radiation Oncology, Affiliated Taizhou Hospital of Wenzhou Medical University, Taizhou, 317000 Zhejiang Province, China; ^2^Department of Pulmonary Medicine, At Enze Hospital, Affiliated Taizhou Hospital of Wenzhou Medical University, Taizhou, 317000 Zhejiang Province, China

## Abstract

**Background:**

Small-molecule tyrosine inhibitor anlotinib which developed in China has been approved as a third-line treatment for patients with small-cell lung cancer (SCLC). Our previous clinical study found that anlotinib combined with S-1 has better short-term ORR than the single-agent anlotinib of SCLC and other small-molecule vascular targeted drug therapies in the treatment of SCLC. However, the molecular mechanism of those effect remains unclear.

**Methods:**

SCLC cell line H446 was treated with either anlotinib, 5-FU alone, or combination. The cellular effects including cell viability, cell apoptosis, cell cycle, cell migration, and invasion were explored to evaluate the cell proliferation level. Western blot was performed to determine the protein levels of the combined action of the two drugs. The xenograft mouse model was established by injection of H446 cells into mouse, and the animals were randomized and assigned for the drug treatments. Body weights and tumor sizes were recorded. WB was conducted using tumor tissues. All data were collected and statistically analyzed using *t*-test to reveal the underlying molecular mechanism.

**Results:**

When anlotinib was combined with 5-FU, the IC50 value of cells was significantly reduced. And apoptosis, cell cycle arrest, and cell motility rates were stronger when anlotinib combined with 5-FU than in the anlotinib or 5-FU alone. In H446 cell-derived xenograft mouse model, tumor volumes were significantly decreased in Anlo/5-FU combination group than anlotinib or 5-FU alone group. Western blot showed the decreasing expression of p-Src/p-AKT in the Anlo/5-FU group.

**Conclusion:**

Our data revealed that the treatment of combination of antitumor angiogenesis agent anlotinib with chemotherapy drug 5-FU may have synergistic cytotoxicity to SCLC in vitro and in vivo. This treatment modality reduced cell proliferation and migration via Src/AKT pathway. This new strategy may be a promising treatment for SCLC but needs to be confirmed in future clinical trials.

## 1. Introduction

Lung cancer (LC) is one of the most common cancers and a leading cause of cancer-related death worldwide [1–3]. About 10%-15% of lung cancer is small-cell lung cancer (SCLC) [4, 5]. Although SCLC comprises a small percentage of the total overall lung cancer cases, it presents as a very aggressive disease, which has a great tendency to metastasize early. The median overall survival (OS) of SCLC is 2–4 months for patients who does not receive any treatment from the time of diagnosis [6]. The therapeutic options for SCLC are limited. In recent years, new targeting drugs for SCLC are also being explored, and substantial progress has been made in the treatment of cancer, but the progress is low [7].

A multitargeting tyrosine kinase inhibitor anlotinib (AL3818) developed independently in China as a third-line treatment for advanced NSCLC [8] and for EC-SCLC in 2019 [9], which targets VEGFR, PDGFR, fibroblast growth factor receptor (FGFR), and c-kit. In addition, anlotinib also inhibits tumor cell proliferation [10]. Phase II and III clinical trials and other basic studies have shown the promising results for anlotinib in the treatment of various type of solid tumors including NSCLC [8], liver cancer [11], renal cancer [12], colorectal cancer [13], and thyroid cancer [14].

5-Fluorouracil (5-FU) is a fluorouracil-containing analogue that was designed as an anticancer drug more than 40 years ago and has been used to treat many cancers including SCLC [15, 16]. 5-FU works mainly by inhibiting thymine synthase activity to prevent the formation of thymine which is needed for DNA synthesis and destroy the structure and function of RNA by abnormal nucleotide incorporation. Thus, 5-FU can damage tumor cells and control the malignant growth [17]. S-1 is a novel type of oral fluorouracil anticancer agent and was used in patients with SCLC. A phase II clinical study in Japan showed a very limited response rate of S-1 monotherapy in the treatment of recurrent SCLC, in which the ORR was only 3.8% [18].

Targeted therapy combined with chemotherapeutic agents can significantly improve the therapeutic effect, such as anlotinib combined with irinotecan exhibited an enhanced effects of reducing cell growth rate and inducing apoptosis in SCLC cell model [19]. In addition, in our previous retrospective study of anlotinib combined with S-1 in the treatment of SCLC, we found that the short-term ORR was 50%, and DCR was 100% in the group of combination, which was much higher than that the treatment along with anlotinib or other single-agent vascular targeting drugs [20]. However, the underlying molecular mechanism is still unclear.

In the present study, we will elucidate the novel mechanism why anlotinib combined with 5-FU has synergistic anticancer effects in the treatment of patients with SCLC by using cell and mouse models and provide evidence for further clinic therapy.

## 2. Material and Methods

### 2.1. Reagents and Cell Culture

Human SCLC cell lines H446, H1688, and H187 were purchased from the Cell Bank of Chinese Academy of Sciences (Shanghai, China) cultured in complete medium RPMI-1640 contained 10% fetal bovine serum and 1% penicillin/streptomycin. The cells were cultured in a humidified incubator with 5%CO_2_ at 37°C. Anlotinib (Nanjing Chia Tai Tianqing Company, Nanjing, China) and 5-FU (purchased from MCE) were diluted in RPMI-1640 medium with different concentration gradients.

### 2.2. Cell Proliferation Ability Assay

The effects of drugs on cell proliferation were determined by CCK8 kit (cat. no. E606335-0500, Sangon Biotech, Shanghai). The cells were plated in 96-well plate at a density of 5000 cells/well and cultured for 24 h, and then, the cells were treated with anlotinib, 5-FU alone, or combination of both at various concentrations for another 72 h. Before detection, 10 *μ*L of CCK8 reagent was added to each well and incubated for 2 h at 37°C. Then, the absorbance was detected by microplate analyzer at the wavelength 450 nm. All experiments were performed three times.

### 2.3. Apoptosis Assay

Annexin V and propidium iodide kit (BD Biosciences, USA) was used to determine cell apoptosis by flow cytometry according to the manufacturer's instructions. The H446 cells treated with alone or both of anlotinib and 5-FU at IC50 levels for 24 h were harvested, washed with PBS, and then stained with annexin V and PI for 15 min at room temperature (RT) in the dark. The samples were immediately detected by a flow cytometry (CytoFLEX, Beckmen).

### 2.4. Cell Cycle Assay

The harvested H446 cells were treated with different drugs and washed twice with cold PBS. After a centrifugation of 1100 g for 5 min at 4°C, removed the supernatant and then added ice-cold 75% ethanol to became a cell suspension at 4°C overnight. The fixed cells were centrifuged again and washed twice with cold PBS. Then, removed supernatant and stained with 500 *μ*L PBS-containing propodeum iodide and RNase A (Meilun Cell Cycle and Apoptosis Analysis Kit, Dalian Meilun Biotechnology Co, Ltd.) at 37°C water bath away from light for 30 min. The samples were detected by flow cytometry.

### 2.5. Analysis of Cell Migration Ability

Logarithmic growth phase cells were taken to adjust cell concentration to 1 × 10^6^ cells per milliliter and then plated in 6-well dishes and cultured overnight. Next day, when the cells grown to 90% density, the cells were scraped with a 200 *μ*L pipette tip and washed twice with PBS after removing the medium; then, added different drugs and incubated overnight at 37°C. Photographed the wound area with a 10× magnification microscope, and the distance of wound movement was measured by comparing the two time periods.

### 2.6. Cell Invasion Assay

The H446 cells were resuspended (1 × 10^6^ cells/ml) and seeded 150 *μ*L in serum-free medium containing 0.1%BSA and Matrigel (1 : 3 dilution; Corning, Inc.) with concentration of anlotinib and 5-FU at IC50 on transwell upper chamber. The medium containing 5% FBS was placed in the lower chamber. The cells at the bottom membrane were fixed with absolute methanol for 15 min and stained with 0.5% crystal violet for 30 min at room temperature after incubation for 24 h. Then, gently wipe the upper chamber side cells with running water to clean. Five fields were randomly selected under a light microscope to count the number of infiltrating cells on the membrane at a magnification of 10×.

### 2.7. Western Blot Analysis

The cells with the aforementioned method were incubated for 24 h. Protein from cells was isolated using lysis buffer. Bicinchoninic acid protein assay kit (BCA, Thermo Fisher Scientific, Inc.) was used to detect protein concentration. Cell lysate and 4× loading buffer in a ratio of 3 to 1 were mixed and boiled at 95°C for 10 min. The loading protein was separated by 10% SDS-PAGE gel and then wet-transferred for 2 hours to a PVDF membrane (EMD Millipore, USA). 5% nonfat milk was used for blocking for 1 h at room temperature and then incubated with primary antibody over night at 4°C. The antibodies used in this study were as follows: AKT (ab179463, Abcam, 1 : 1000), pAKT (ab38449, Abcam, 1 : 2000), GAPDH (ab181602, Abcam, 1 : 5000), Src (2109S, CST, 1 : 1000), pSrc (2105S, CST, 1 : 1000), pVEGFR (3370S, CST, 1 : 1000), and VEGFR2 (9698S, CST, 1 : 1000); the membrane was washed with PBST three times, 15 minutes each, and then incubated with secondary antibody for 1 h at room temperature. SuperSignal West Pico chemiluminescence substrate was used to enhance chemiluminescence detection of relevant protein bands. FluorChem HD2 software was used for analysis and quantification of Western blot.

### 2.8. Xenograft Mouse Model

Male BALB/c-nu mice (4 to 6 weeks of age, total = 24) were purchased from Shanghai SLK Laboratory Animal Company. 1 × 10^6^ NCI-H446 cells were injected subcutaneously into the right flank of mice. The graft volume was calculated by the following standard formula: length × width × width × 0.5. When the tumors reached about 150 mm^3^, mice were randomly divided into four groups (*n* = 6, per group). Group 1 was treated with oral saline, and group 2 was treated by intragastrically with 1.5 mg/kg anlotinib once daily, and group 3 was treated by intraperitoneal injection with 100 mg/kg 5-FU per week, and group 4 was treated with both of anlotinib and 5-FU. The health status and tumor growth of mice were observed every day. Tumor volume and mouse weight were recorded every 3 days. After 14 days of drug intervention, mice were given oral normal saline for 7 days. After the mice were sacrificed by dislocation, the tumor tissue was taken, the weight and volume of the tissue were measured, and the tumor tissue was separated and fixed with liquid nitrogen.

### 2.9. Statistical Analysis

All data were analyzed by GraphPad Prism 6 (GraphPad Software, Inc., USA) statistical software. The results were reported as mean ± SD. The differences between groups were statistically analyzed by one-way ANOVA. All *P* values were based on *t*-test statistical analysis. *P* < 0.05 was considered as statistically significant difference.

## 3. Result

### 3.1. Anlotinib Combined with 5-FU Inhibits Cell Growth in Small-Cell Lung Cancer Cells

**Figure 1:**
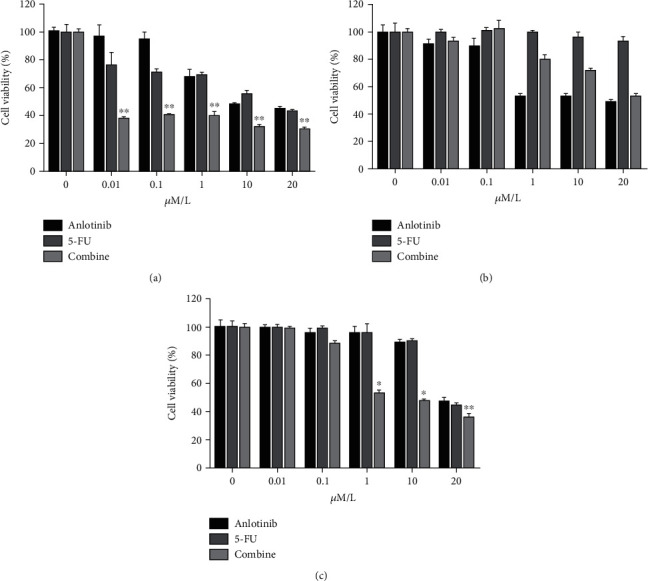
Effect of anlotinib combined with 5-FU in SCLC cell lines. The H446, H1688, and H187 cells were treated with anlotinib, 5-FU, or combination of both for 72 h, and cell growth was detected using CCK8 kit. The inhibition rates in the (a) H446 cells, (b) H1688 cells, and (c) H187 cells following treatment with anlotinib and 5-FU in different drug concentrations (0 *μ*M, 0.01 *μ*M, 0.1 *μ*M, 1 *μ*M, 10 *μ*M, and 20 *μ*M) while 5-FU remained a fixed concentration in the mixture. The bars represent mean ± SEM of the three independent experiments. ^∗^*P* < 0.05 and ^∗∗^*P* < 0.01 compared with anlotinib. SCLC: small-cell-lung cancer.

To verify the toxicity of the two drugs in small-cell lung cancer, we chose some SCLC cell lines including H187, H1688, and H446. We fixed 5-FU concentration to IC50 value in the combination of the two drugs when anlotinib was in a gradient concentration. After 72 hours of drug action in the H446 cells, we measured the IC50 value of anlotinib which was 8.232 *μ*M as 5-FU was 15.32 *μ*M. In the H187 cells, the IC50 values of anlotinib and 5-FU were 20.47 *μ*M and 22.4 *μ*M. CCK-8 assay exhibited that anlotinib, 5-FU, or the combination of both in H446 and H187 cell lines has inhibitory effect which was not obvious in the H1688 cell line, and the cells were dose-dependently suppressed (Figures 1(a)–1(c)).

### 3.2. Anlotinib Combined with 5-FU Promoted Apoptosis in Small-Cell Lung Cancer Cells

**Figure 2:**
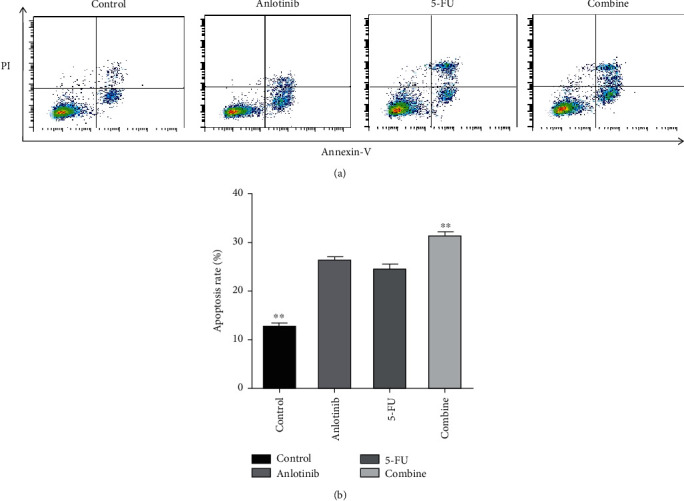
Anlotinib combined with 5-FU induces cell apoptosis in the H446 cells. The cells were treated with anlotinib and 5-FU or combination of both for 24 hours. Apoptosis determined by annexin V-FITC/PI staining by flow cytometry. (a) The H446 cells were treated with anlotinib (5 *μ*M) and 5-FU (10 *μ*M) or combination of both. (b) The apoptosis rate (%) included Q3 and Q4. All experiments were repeated three times. The bars represent mean ± SEM of the three independent experiments. ^∗^*P* < 0.05 and ^∗∗^*P* < 0.01 compared with anlotinib.

According to the result of cell proliferation assay, we chose the concentration of IC50 for two drugs to further explored the cytotoxic mechanism of anlotinib combined with 5-FU. We evaluated the effect of both two drugs on cell apoptosis by flow cytometry in H446 cell line. The cells were treated with anlotinib (5 *μ*M) and 5-Fu (10 *μ*M) for 24 h. According to the results, apoptosis was increased in the anlotinib or 5-FU treatment groups compared with the control group (Figure 2(a)). More significantly, combination therapy inhibited apoptosis more effectively than monotherapy (Figure 2(b)) (*P* < 0.01).

### 3.3. Anlotinib Combined with 5-FU Induced Cell Cycle Arrest in the H446 Cells

**Figure 3:**
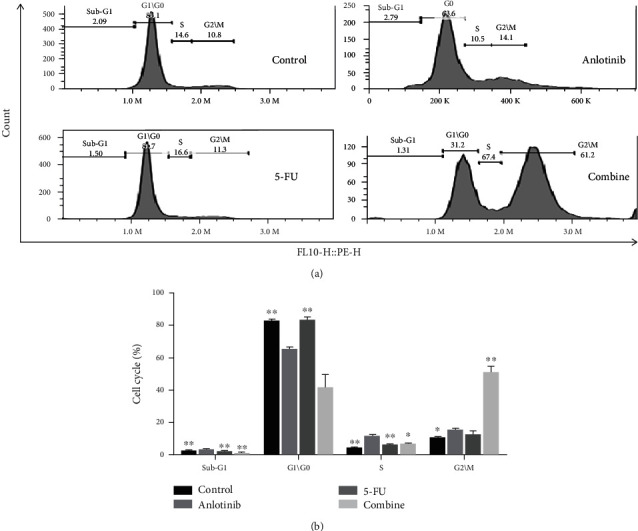
Anlotinib combined with 5-FU induced cell cycle arrest in the H446 cells using cell cycle kit. (a) The H446 cells were treatment with anlotinib (5 *μ*M) and 5-FU (10 *μ*M) or combination of both for 24 h. (b) The cell cycle rate (%) including G1\G0, S and G2\M. All experiments were repeated three times. The bars represent mean ± SEM of the three independent experiments.^∗^*P* < 0.05 and ^∗∗^*P* < 0.01 compared with anlotinib.

Flow cytometry was performed to investigate the effect of anlotinib (5 *μ*M) or 5-FU (10 *μ*M) or both of two for 24 h on the cell cycle. The results demonstrated that the combination group significantly induced cell cycle arrest (Figure 3(a)), with an increased number of cells in the G2/M phase compared with control and single-drug treatment group (Figure 3(b)).

### 3.4. Anlotinib and 5-FU Reduced Migration and Invasion Abilities

**Figure 4:**
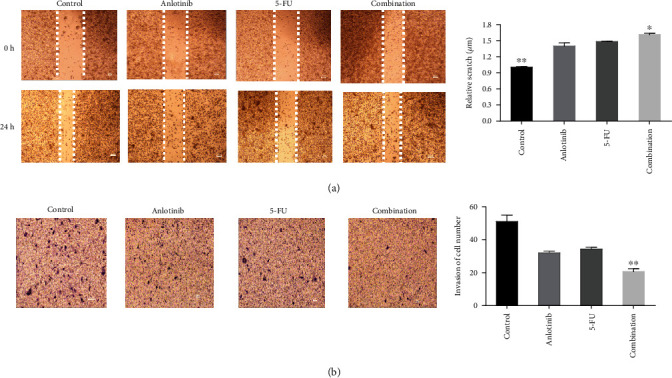
Anlotinib combined with 5-FU inhibits migration and invasion in the H446 cells. And the cells were fixed in 20% paraformaldehyde and stained with 0.5% crystal violet. (a) The effect of anlotinib (5 *μ*M) and 5-FU (10 *μ*M) or combination of both on cell migration evaluated by wound healing methods in the H446 cells. (b) The effect of anlotinib (5 *μ*M) and 5-FU (10 *μ*M) or combination of both on cell invasion evaluated by transwell assays in the H446 cells. The results are from the three independent experiments.^∗^*P* < 0.05 and ^∗∗^*P* < 0.01 compared with anlotinib.

To determine the possible effect of anlotinib and 5-FU on the metastatic potential of small lung cancer cells, the *in vitro* wound healing and transwell assays were performed. The H446 cells were treated with anlotinib (5 *μ*M) and 5-FU (10 *μ*M) or combination of both of the two treatments for 24 h to observe the ability of cell migration and invasion. The directed mobility of untreated cells was higher than that of anlotinib, 5-FU, or both as shown in Figure 4(a). It was very clear that the combination of the two drugs inhibited more cell migration compared to anlotinib or 5-FU alone. Similarly, the transwell experiment showed that the combination group was less invasive than the group alone and the control group (Figure 4(b)).

### 3.5. Anlotinib and 5-FU Acted through the Src/AKT Pathway

**Figure 5:**
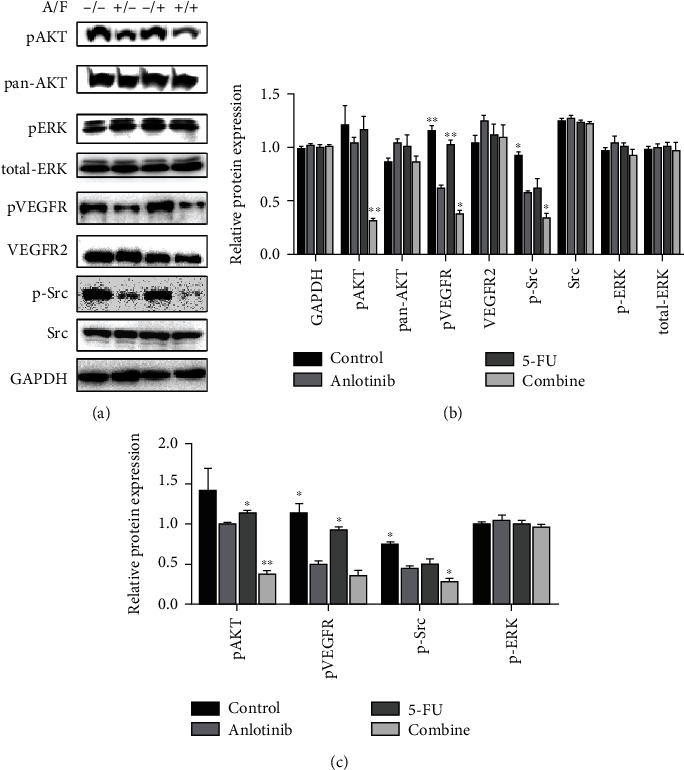
Anlotinib combined with 5-FU affected cell proliferation through Src/AKT pathway in the H446 cells through Western blot. (a)p -AKT/pan-AKT, p-ERK/total ERK, pVEGFR/VEGFR2 p-Src/Src, and GAPDH in each treatment group were tested by Western blot. (b) The relative protein expression of which relative to GAPDH was analyzed by GraphPad Prism. (c) The relative protein expression of pAKT/pERK/pVEGFR/p-Src relative to pan-AKT/total-ERK/VEGFR2/Src was analyzed by GraphPad Prism. The results are from the three independent experiments.^∗^*P* < 0.05 and ^∗∗^*P* < 0.01 compared with anlotinib.

The Src/AKT is a key protein in an angiogenic pathway which played an important role in the invasion and migration of cells. To test our hypothesis, we used Western blot to detect the expression levels of the key proteins in the relevant signaling pathways (Figure 5(a)). The H446 cells were treated with anlotinib (5 *μ*M) and 5-FU (10 *μ*M) or combination treatment for 24 h. Then, we analyzed the relative expression of the protein (Figure 5(b)), and at the same time, pAKT, pVEGFR, and pSrc were both inhibited in combination treatment group (Figure 5(c)).

### 3.6. Anlotinib and 5-FU ﻿Affect Tissue Proliferation and the Expression of Relative Protein by Western Blot

**Figure 6:**
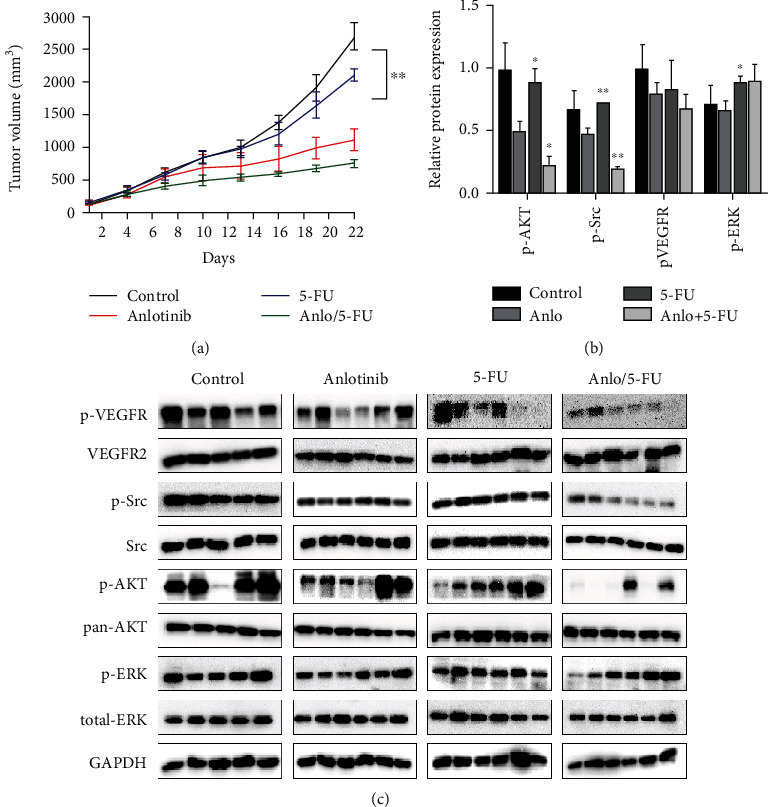
Anlotinib combined with 5-FU affected cell proliferation and angiogenesis in vivo. 24 male BALB/c-nu mice were randomly divided into four groups treated with oral saline, 1.5 mg/kg anlotinib, 100 mg/kg 5-FU, or both of anlotinib and 5-FU. (a) Tumor volume in mice after 21-day treatment. (b) The protein expression for each group. (c) The relative protein expression levels of each group. The results are from three independent experiments. ^∗^*P* < 0.05 and ^∗∗^*P* < 0.01 compared with anlotinib.

After cell experiment *in vitro*, we then used mice for tumor tissue expression. As shown in Figure 6(a), the combination of the two drugs suppressed tumor growth more than the other three groups. Western blot was used to verify the results *in vitro* in Figures 6(b) and 6(c); the protein expression was analyzed by Western blot and showed that the expression of pVEGFR, p-AKT, and p-Src for the group of anlotinib combined with 5-FU was lower than the other three groups (*P* < 0.05).

## 4. Discussion

Lung cancer is a major public health problem because of its high morbidity and mortality rates and high recurrence rates [21]. Treatment strategies for SCLC have not progressed in the past four decades, with first-line standard care. Therefore, the search for a new antitumor treatment is still an urgent clinical problem. Anlotinib is approved by Chinese Food and Drug Administration (CFDA) for indication of NSCLC in 2018 based on the ALTER 0303 study [8], which could inhibit both tumor angiogenesis, cell proliferation, migration, and invasion in different tumor cells [10, 22, 23]. In an ALTER 1202 clinical trial, anlotinib, as a third-line treatment for SCLC, has shown similar efficacy in NSCLC, prolonging progression-free survival (PFS) and OS [8]. However, the ORR of objective response rate was only 4.9%, and the OS was only prolonged by 3 months. In solid tumors, anlotinib significantly reduced cell viability of SCLC cell lines, including proliferation, migration, and invasion of the H446 cells [24], which also occurred in colorectal cancer in 5-FU-resistant cells [25].

In this study, we found that anlotinib combined with 5-FU could significantly inhibit the proliferation of the H446 cells *in vitro a*ccording to Figures 1 and 2. We found that anlotinib interferes with the normal cell cycle and influences H446 cell proliferation according to Figures 3 and 4. We showed that anlotinib combined with 5-FU could increase the expression of both pAKT and pSrc according to Figure 5. And *in vivo*, we found that anlotinib combined with 5-FU significantly inhibit tumor growth, and the expressions of some key protein were also inhibited according to Figure 6. All of the above phenomena showed that the effect of anlotinib combined with 5-FU was consistent with the clinical effect trend of the combination of the two drugs [26]. According to Figures 1–6, combination of anlotinib and 5-FU could inhibit H446 cell migration and angiogenesis.

Classic AKT/ERK proteins and their pathways play a key role in multiple cellular functions including proliferation, adhesion, migration, invasion, metabolism, and survival [27], as well as a regulatory role in angiogenesis [28]. However, we did not see a significant change in ERK/p-ERK (Figure 5). Src kinases belong to a family of nonreceptor, intracellular protein tyrosine kinases: Src is activated by autophosphorylation of Tyr416 and can be induced by TGF-*β*1 and epidermal growth factor (EGF) [29]. The Src plays a key role in tumor proliferation and progression via migration, invasion, and angiogenesis [30]. Moreover, its downstream AKT signaling pathway plays a role in inhibiting SCLC cell proliferation [31], which is consistent with our results. And previous studies have showed that 5-FU inhibits cell proliferation via AKT signal pathway [32, 33].

In conclusion, the current study demonstrated that anlotinib combined with 5-FU could inhibit SCLC cell proliferation and induce apoptosis and inhibit angiogenesis via Src/AKT pathway. Overall, we preliminarily explored the role of the two drugs in SCLC which is consistent with clinical trial results and confirmed the effect of anlotinib combined with 5-FU in SCLC and provided explanations for clinical phenomena and future therapeutic directions for SCLC.

## 5. Conclusion

Our data revealed that the treatment of combination of antitumor angiogenesis agent anlotinib with chemotherapy drug 5-FU may have synergistic cytotoxicity to SCLC in vitro and in vivo. This treatment modality reduced cell proliferation and migration via Src/AKT pathway. This new strategy may be a promising treatment for SCLC but needs to be confirmed in future clinical trials.

## Data Availability

The research data used to support the findings of this study are included within the article.
